# Rabies Virus Maintained by Dogs in Humans and Terrestrial Wildlife, Ceará State, Brazil

**DOI:** 10.3201/eid1212.060429

**Published:** 2006-12

**Authors:** Silvana R. Favoretto, Cecília C. de Mattos, Nélio B. de Morais, Maria Luíza Carrieri, Benedito N. Rolim, Lucia M. Silva, Charles E. Rupprecht, Edison L. Durigon, Carlos A. de Mattos

**Affiliations:** * Instituto Pasteur de São Paulo, São Paulo, Brazil;; † Universidade de São Paulo, São Paulo, Brazil;; ‡ Centers for Disease Control and Prevention, Atlanta, Georgia, USA;; § Secretaria Estadual de Saúde do Ceará, Fortaleza, Brazil

**Keywords:** Rabies, Brazil, zoonosis, epidemiology, infectious diseases, virology, phylogeny, wildlife, domestic animals

## Abstract

Rabies viruses circulating in Ceará, Brazil, were identified by molecular analysis to be related to variants maintained by dogs, bats, and other wildlife. Most of these viruses are associated with human rabies cases. We document the emergence of a rabies virus variant responsible for an independent epidemic cycle in the crab-eating fox (*Cerdocyon thous*).

After dog rabies control programs were implemented in Ceará State, Brazil, a the number of human cases decreased (*1**,**2*). Thereafter, the epidemiologic importance of rabies in wildlife became evident. From 1990 through 2005, a total of 173 cases of rabies were reported in Cerdocyon thous (crab-eating fox), 25 in Callithrix. j. jacchus (common marmoset) and 6 in Procyon cancrivorous (crab-eating raccoon). During this period, in 13 of 40 human cases reported in Ceará, wildlife was the source of infection (*2*).

In 1996, because of this new epidemiologic situation, public health authorities launched an educational program, and no human cases due to wildlife were recorded in 1999, despite 84 cases in wildlife registered that year (*2*). The objective of this study was to elucidate some of the epidemiologic events involved in rabies emergence among wildlife in Ceará.

## The Study

We studied 22 samples, from dogs, cattle, wildlife, and humans in Ceará, obtained from 1997 to 2003 (Table). Samples were antigenically characterized by using a monoclonal antibody (MAb) panel against the viral nucleoprotein (*3**–**5*). Isolates were injected into the brains of suckling mice, and brain impressions were made for MAb typing (*3**,**5*). Characterization of the samples identified 4 antigenic variants. Antigenic variant-2 (AgV2), maintained by dogs, was found in all C. thous, P. crancrivourous, and human cases and in all dog isolates with the exception of brdg5360, which was positive with all the MAbs. Antigenic variant-3 (AgV3), epidemiologically associated with vampire bats, Desmodus rotundus, was identified in 3 bovine samples. A previously reported profile, representing an AgV that circulates in marmosets in Ceará (*5*), was detected in sample brsg5696.

**Table:** Identification, origin, and antigenic and genetic variant of 22 rabies virus samples isolated from Ceará State, Brazil

Identification	Animal species	Year of isolation	Origin	Antigenic variant	Genetic variant	Group	GenBank accession no.
Brhm4531	Human	1997	Fortaleza	AgV2	Dog	B	DQ447947
Brcth4122	*Cerdocyon thous*	1998	-	AgV2	Dog	B	DQ447948
Brhm5325	Human	2000	Caucaia	AgV2	Dog	C	DQ447949
Brdg5360	Dog	2000	Caucaia	All+	Dog	C	DQ447950
Brcth5361	*C. thous*	2000	Paracuru	AgV2	Dog	B	DQ447951
Brbv5339	Bovine	2000	Antonina do Norte	AgV3	Vampire bat	D	DQ447952
Brbv5374	Bovine	2000	Quixere	AgV3	Vampire bat	D	DQ447953
Brhm5691	Human	2001	Caucaia	AgV2	Dog	C	DQ447954
Brdg5693	Dog	2001	Maranguape	AgV2	Dog	A	DQ447955
Brcth5695	*C. thous*	2001	Barroquinha	AgV2	Dog	B	DQ447956
Brcth5697	*C. thous*	2001	Caninde	AgV2	Dog	B	DQ447957
Brcth5692	*C. thous*	2001	Maranguape	AgV2	Dog	B	DQ447958
Brpcr5698	*P. cancrivorous*	2001	Maranguape	AgV2	Dog	B	DQ447959
Brbv5694	Bovine	2001	Aquiraz	AgV3	Vampire bat	D	DQ447960
Brsg5696	*C. j. jacchus*	2001	Caucaia	AgV new*	Marmoset	E	DQ447961
Brhmu138	Human	2002	Fortaleza	AgV2	Dog	C	DQ447962
Brhmu142	Human	2003	Fortaleza	AgV2	Dog	C	DQ447963
Brhmu130	Human	2003	Umirim	AgV2	Dog	C	DQ447964
Brhmu146	Human	2003	Fortaleza	AgV2	Dog	C	DQ447965
Brhmu145	Human	2003	Tururu	AgV2	Dog	C	DQ447966
Brhmu129	Human	2003	Fortaleza	AgV2	Dog	C	DQ447967
Brhmu131	Human	2003	Maracanau	AgV2	Dog	C	DQ447968

*Pattern related to isolates from marmosets.

**Figure 1:**
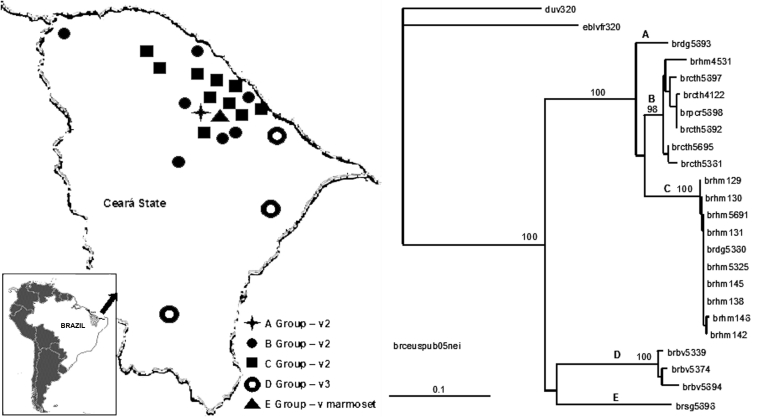
Rabies virus isolates by geographic localization and neighbor-joining tree showing a comparison of the groups formed by Ceará State, Brazil, samples isolated from 1997 to 2003. Bootstrap values of >50% obtained from 100 resamplings of the data using distance matrix methods are shown in the nods.

The Ceará viruses were analyzed genetically through a comparative phylogenetic study based on a 320-bp fragment of the nucleoprotein gene, from position 1157 to 1476, as compared with SADB19 (*5**–**8*). These isolates were also compared with rabies virus variants circulating among domestic animals and wildlife from the Americas. The viral RNA was extracted from infected tissues, and the cDNA was obtained by reverse transcription–PCR techniques, using primers 21 g and 304, and was sequenced with primer 304 (*7**,**9*). The phylogenetic analyses were made by using the PileUp program of the Wisconsin Package Version 10.1 (*10*) and the programs DNADIST, NEIGHBOR, SEQBOOT, and CONSENSE of the PHYLIP package (*11*). The expressed percentages of identity refer to the nucleotide sequences. The trees were obtained with the TREEVIEW program (*12*). The phylogenetic analyses showed segregation in 5 lineages, A–E (Figure 1), which was statistically supported by high bootstrap values.

Lineage A was represented by a sample from a dog from Maranguape, which was obtained in 2001. This virus showed its closest genetic relationship with lineage B (identity 92.4%–94.2%). Lineage B was formed by all the C. thous isolates, a sample from a human bitten by a P. cancrivorous raccoon in Fortaleza in 1997, and a virus from a P. crancrivorous raccoon collected in Maranguape during 2001 (intrinsic identity 96.5%–100%). This lineage showed its highest percentage of identity with lineage C (intrinsic identity 90.6%–92.8%). Lineage C consisted of 9 human samples collected in 5 different counties from 2000 to 2003 and an isolate obtained from a dog in 2000. The samples were highly homologous (intrinsic identity 99.1%–100%). Lineage D included 3 bovines collected in 3 geographically distant counties during 2000 and 2001 (intrinsic identity 97.5%–98.4%). Lineage E was represented by the only sample collected from a C. j jacchus marmoset, These last 2 lineages were related distantly to all the others.

**Figure 2:**
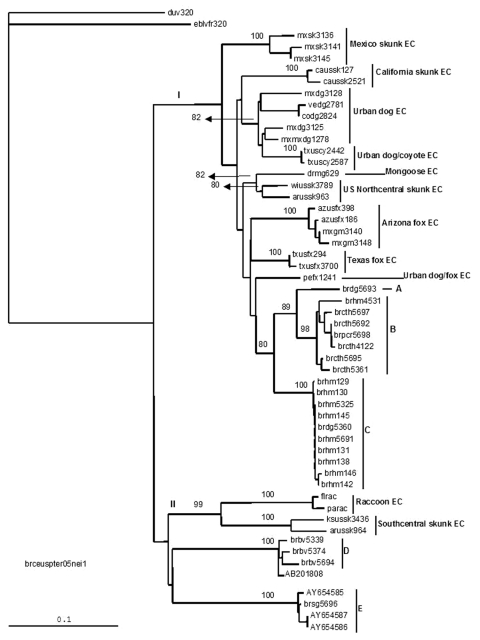
Neighbor-joining tree showing a comparison of Ceará samples (groups A, B, C, D, E) with isolates obtained from the Americas. Bootstrap values of >50% obtained from 100 resamplings of the data using distance matrix methods are shown in the nods. The sequences from Latin America used in the comparison were identified as as follows: group I, dogs and terrestrial wildlife from Mexico, Venezuela, Colombia, Dominican Republic, and Peru (mxsk, skunk from Mexico; mxdg and mxmx, dog from Mexico; vedg, dog from Venezuela; codg, dog from Colombia; drmg, mongoose from Dominican Republic; mxgm, bobcat from Mexico; pefx, fox from Peru) and terrestrial wildlife from the United States (caussk, skunk from California; txuscy, coyote from Texas; wiussk, striped skunk from Wisconsin; arussk, striped skunk from Arkansas; azusfox, gray fox from Arizona; txusfx, gray fox from Texas); and group II, terrestrial wildlife from the United States (flrac, raccoon from Florida; parac, raccoon from Philadelphia; ksussk, striped skunk from Kansas; arussk, striped skunk from Arkansas). The antigenic variant and endemic cycle to which it belongs are shown in the tree. (GenBank accession no. AB201803 is a vampire bat from Brazil and nos. AY654585, AY654587, AY654586 are humans and a marmoset from Brazil). EC, endemic cycle.

When compared with representatives of rabies variants maintained by terrestrial and bat species in the Americas (Figure 2), lineages A, B, and C continue to segregate as independent lineages with high statistical support. Ceará bovine samples representing lineage D clustered with D. rotundus and D. rotundus–related cases from Latin America (intrinsic identity 94%–97.8%).

The only sample representative of lineage E segregated with 2 isolates from humans bitten by C. j. jacchus and a sample collected from a marmoset kept as a pet (*5*). The isolates were highly homologous to each other (intrinsic identity 98.7%–100%).

## Conclusions

A thorough description of rabies epidemiology depends on a comprehensive surveillance program and application of accurate molecular methods to discriminate among different variants and the emergence of new foci. Antigenic and limited sequencing analyses were used to better understand the emergent epidemiologic events in wildlife in Ceará, Brazil. These analyses allowed identification of 5 potential cycles in this region, despite antigenic homogeneity.

Lack of antigenic and genetic relationships of sample brdg5693, representing lineage A, with the rest of the isolates from Ceará and the known terrestrial rabies vectors from the Americas shows that this virus is a variant not previously described. This virus was geographically and temporarily associated with samples brpcr5698 and brcth5692, obtained in Maranguape during 2001. These circumstances demonstrated the existence of at least 2 overlapping endemic cycles in this area. Lineage B was formed mainly by isolates from C. thous, which indicates the existence of an emerging rabies cycle in this species.

The epidemiologic situation in Ceará was complicated because of overlapping distributions of dog and C. thous rabies cases (Figure 1). Tree topology and genetic relationships between dog and C. thous variants suggested that the canine virus was introduced in C. thous populations because of spillover events, which gave rise to an emergent cycle. A similar event was described between domestic dogs and Canis adustus (jackal) in Zimbawe. In this case, the variant circulating in dogs was introduced into the C. adustus population by spillover events, with the consequent emergence of an independent cycle (*13*). Recently, the hoary fox has been identified as a rabies reservoir in Brazil (*14*).

Inclusion in lineage B of an isolate obtained from a human bitten by a P. cancrivorous raccoon and another sample collected from this species suggested the risk of establishing C. thous variant in P. cancrivorous. The niches of these 2 species overlap, which facilitates their encounters. Additional surveillance is necessary to clarify this situation.

Epidemiologic data which indicates that humans had been exposed to dog bites, results of molecular characterization, and inclusion of a dog isolate in the C lineage strongly incriminate the dog as the reservoir of this variant. Identification of the source of infection by using classic surveillance alone is complicated by the presence of multiple cycles of transmission. Genetic comparison of samples from lineage D with viruses representing bats viruses from the Americas helped to identify D. rotundus as the source of livestock infection.

The close genetic relationship of sample brsg5696 with rabies isolates obtained from C. j. jacchus and human cases bitten by marmosets further supported C. j. jaccuss as the most important vector of this variant. This finding indicates that this species plays an important role for disease maintenance in nature.

Methods for antigenic and genetic identification of rabies samples isolated in the Americas have contributed effectively to the development of health programs, as well as recognition of possible wild reservoirs of urban rabies. The emergence of new cycles in Latin American wildlife indicates the need to strengthen surveillance programs in these species and research development for the evaluation of the feasibility of oral vaccination interventions.
